# MDA5 complements TLR3 in suppression of neuroblastoma

**DOI:** 10.18632/oncotarget.4511

**Published:** 2015-07-09

**Authors:** Wen-Ming Hsu, Chao-Cheng Huang, Hsin-Yu Lee, Pei-Yi Wu, Min-Tsui Wu, Hui-Ching Chuang, Li-Ling Lin, Jiin-Haur Chuang

**Affiliations:** ^1^ Department of Surgery, National Taiwan University Hospital and National Taiwan University College of Medicine, Taipei, Taiwan; ^2^ Department of Pathology, Kaohsiung Chang Gung Memorial Hospital, Chang Gung University College of Medicine, Kaohsiung, Taiwan; ^3^ Department of Life Science and Institute of Zoology, National Taiwan University, Taipei, Taiwan; ^4^ Department of Pediatric Surgery, Kaohsiung Chang Gung Memorial Hospital, Chang Gung University College of Medicine, Kaohsiung, Taiwan; ^5^ Department of Otolaryngology, Kaohsiung Chang Gung Memorial Hospital, Chang Gung University College of Medicine, Kaohsiung, Taiwan; ^6^ Department of Medical Research, Kaohsiung Chang Gung Memorial Hospital, Chang Gung University College of Medicine, Kaohsiung, Taiwan

**Keywords:** MDA5, neuroblastoma, poly(I:C), RIG-I, TLR3

## Abstract

Toll-like receptor3 (TLR3) has been confirmed to be differentially expressed in neuroblastoma (NB), and predicts a favorable prognosis with a high expression in tumor tissues. Treatment with TLR3 agonist - polyinosinic-polycytidylic acid [poly(I:C)] could induce significant but limited apoptosis in TLR3-expressing NB cells, suggesting that other viral RNA sensors, including melanoma differentiation-associated gene 5 (MDA5) and retinoic acid-inducible gene-I (RIG-I) in the cytosolic compartment might also be implicated in poly(I:C)-induced NB cell death. MDA5 and RIG-I were induced by poly(I:C) to express in two of six NB cell lines, SK-N-AS (AS) and SK-N-FI, which were associated with up-regulation of caspase9 and active caspase3. While knockdown of either MDA5 or RIG-I alone is ineffective to decrease caspase9 and active caspase3, simultaneously targeting MDA5 and TLR3 showed the best effect to rescue poly(I:C) induced up-regulation of mitochondrial antiviral signaling protein (MAVS), caspase9, active caspase3, and apoptosis in AS cells. Over-expression of MDA5 in FaDu cells resulted in significantly less colony formation and more poly(I:C)-induced cell death. Further studies in human NB tissue samples revealed that MDA5 expression in NB tissues predicted a favorable prognosis synergistically with TLR3. Our findings indicate that MDA5 may serve as a complementary role in the TLR3 activated suppression of NB.

## INTRODUCTION

Neuroblastoma (NB) is a pediatric solid tumor characterized by its wide range of clinical manifestations and poor outcome for those with high-risk clinical phenotypes, despite significant advance in the treatment in the last 10 years [1, 2]. Toll-like receptors (TLRs) are initiators of the innate immune response to pathogens. One of the TLR family members - TLR3 has been confirmed to be differentially expressed in NB, and predict a favorable prognosis with a high expression in tumor tissues by our group [3, 4]. TLR3 agonist polyinosinic-polycytidylic acid [poly(I:C)] could induce apoptosis in TLR3-expressing NB cells, preferentially through mitochondrial pathway [5]. However, the effects of poly(I:C) on NB cell apoptosis through TLR3 is limited, suggesting that other cellular mechanisms in addition to TLR3 should participate in the poly(I:C) induced cell death.

In addition to TLR3, there are other viral RNA sensors in the cells, including melanoma differentiation-associated gene 5 (MDA5) and retinoic acid-inducible gene-I (RIG-I), which have been implicated in mitochondrial apoptosis of human melanoma cells, as well as proimmunogenic apoptosis of human ovarian cancer cells [6, 7]. MDA5 was initially isolated by subtraction hybridization as a gene with interferon-inducible putative RNA helicase, which demonstrated double-stranded RNA-dependent ATPase activity and melanoma growth-suppressive properties [8]. RIG-I was isolated by differential display-PCR from acute promyelocytic leukemia cells treated with *all-trans* retinoic acid, and initially known as RIG-E [9].

Both MDA5 and RIG-I belong to retinoic acid-inducible gene-I-like receptor (RLR) family and function uniquely as DExD/H-box helicases for anti-viral innate immunity [10]. While TLR3 is located on the endosomes, MDA5 and RIG-I are cytoplasmic RNA sensors [10]. Upon stimulation of the cells with either viral infection or intrinsic/extrinsic nucleic acids, MDA5 and RIG-I could interact with mitochondrial antiviral signaling protein (MAVS) [also known as IFN-beta promoter stimulator (IPS)-1], which activates signaling pathways of NFkappaB and interferon regulatory factors (IRFs) to trigger apoptosis of cancer cells [11, 12].

Recently it was shown that targeting MDA5 and RIG-I could effectively induce apoptotic signaling and counteract cancer cell heterogeneity in glioblastoma [13], suggesting that MDA5 and RIG-I might also be implicated in the suppression of another neurogenic cancer. In this study, we found that upon treatment with poly(I:C), SK-N-AS (AS) and SK-N-FI NB cells showed an increase of MDA5 and RIG-I expression levels, which was associated with an increased expression of caspase9 and active caspase3. In addition, only simultaneously targeting MDA5 and TLR3 showed the best effect to rescue poly(I:C)-induced cancer cell suppression. Further studies of human NB tissue samples also revealed that MDA5 expression in NB tissues predicted a favorable prognosis synergistically with TLR3. These results indicated a complementary role of MDA5 in TLR3 agonist treatment of NB.

## RESULTS

### Differential expression of dsRNA sensors, caspase9 and caspase 3 in NB cell lines after poly(I:C) treatment

**Figure 1:**
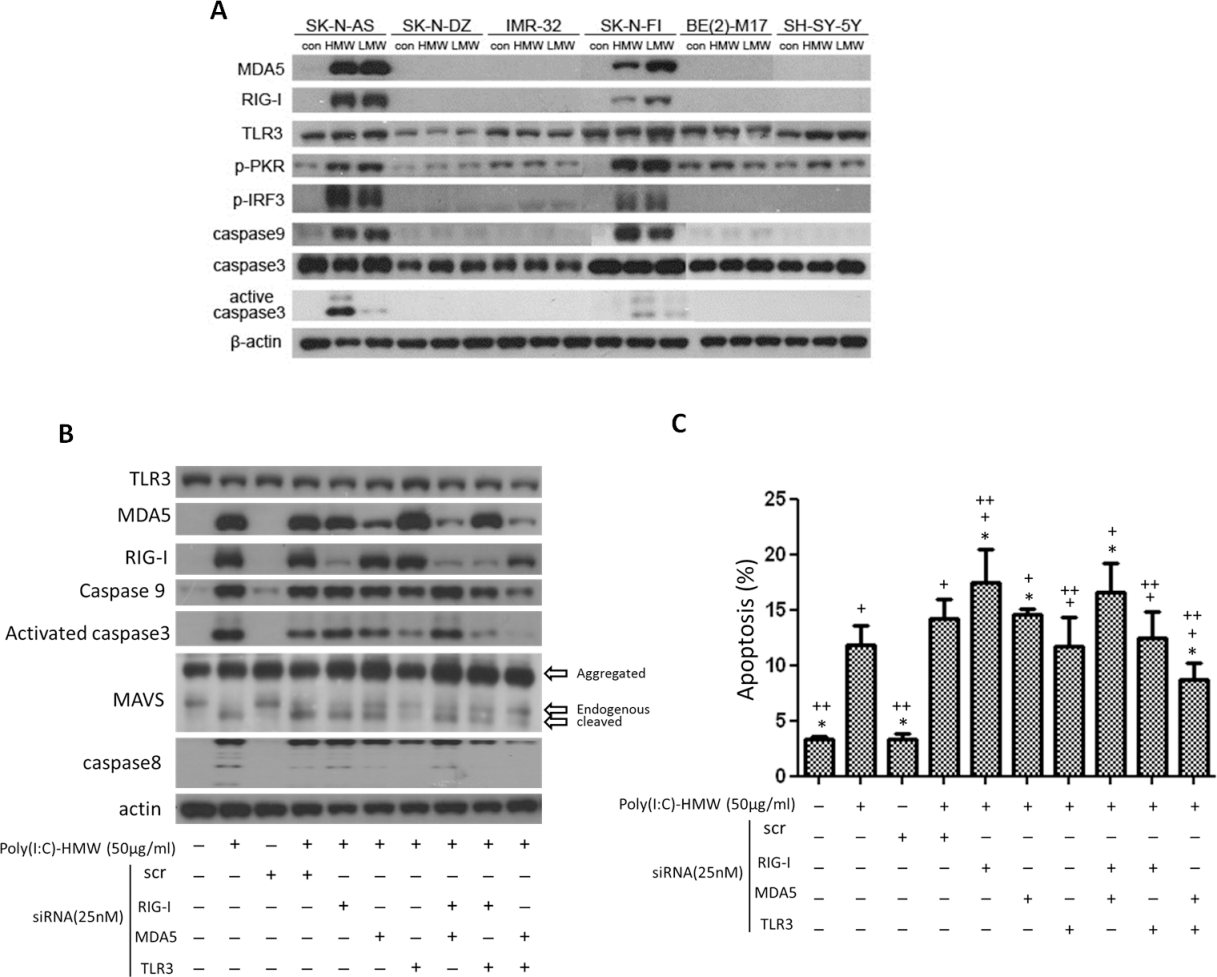
Differential expression of MDA5, RIG-I and TLR3, downstream signaling molecules, and cell apoptosis after treatment with poly(I:C) and siRNA **A.** Up-regulation of MDA5 and RIG-I, as well as p-PKR, p-IRF3, caspase9 and active caspase3 was only observed in SK-N-AS (AS) and SK-N-FI (FI), but not in the other 4 cell lines after treatment with either high molecular weight (HMW) or low molecular weight (LMW) poly(I:C). **B.** In SK-N-AS cells, only concomitant knockdown of MDA5 and TLR3 were more effective to suppress upregulation of caspase9 and activated caspase 3 triggered by poly(I:C) treatment than either single knockdown or other double knockdowns of TLR3, MDA5, and RIG-I. Cleaved MAVS and caspase8 were also most significantly decreased in double knockdown of MDA5 and TLR3 **C.** Apoptosis measured by PI staining and flow cytometry in SK-N-AS cells after treatment with poly(I:C) and single or concomitant knockdown of TLR3, MDA5, and RIG-I. scr: scrambled siRNA. *p < 0.05 vs. Poly(I:C), +*p* < 0.05 vs. scr, ++*p* < 0.05 vs. scr+poly(I:C).

Among the six cell lines, SK-N-AS, SK-N-FI, and SH-SY5Y had a normal copy number of *MYCN*, while SK-N-DZ, IMR-32 and BE(2)-M17 had *MYCN* amplification. All NB cell lines expressed a base-line level of TLR3, although the level was low in SK-N-DZ. After treatment with poly(I:C), there was no significant change of TLR3 protein expression in SK-N-DZ, IMR-32 *BE(2)-M17*, and SK-N-AS, while there was mild up-regulation of TLR3 expression in, SK-N-FI and SH-SY-5Y. None of the wild-type cell lines expressed MDA5 and RIG-I. But after high or low molecular weight poly(I:C) treatment, SK-N-AS and SK-N-FI showed a significant up-regulation of MDA5 and RIG-I. (Figure 1A). Interestingly, there was a significant increase of TLR3 mRNA in SK-N-AS after poly(I:C) treatment, despite no simultaneous increase of TLR3 protein levels. (Supplementary Figure 1A)

Phosphorylated protein kinase R (p-PKR), an interferon-induced, double-stranded RNA-activated protein kinase, was also activated by both high or low molecular weight poly(I:C) in both SK-N-AS and SK-N-FI, with an up-regulation of phosphorylated interferon regulatory factor 3 (p-IRF-3) (Figure 1A). Subsequent to the up-regulation of dsRNA sensors and their adaptor molecules, there were up-regulation of caspase9 and activated caspase3 in both SK-N-AS and SK-N-FI, but not in other cell lines (Figure 1A). Since expression of MDA5 and RIG-I, as well as activated caspase-3 was consistently higher in SK-N-AS than in SK-N-FI after treatment with high molecular weight (HMW) poly(I:C), the results of subsequent *in vitro* studies were based on the findings in SK-N-AS.

### Expression of PKR, IRF3, MAVS, caspase 9/3 and apoptosis in SK-N-AS after poly(I:C) treatment and siRNA targeting TLR3, MDA5 and RIG-I

Treatment of SK-N-AS cells with poly(I:C)-HMW resulted in significant increase of mRNA levels of TLR3, MDA5 and RIG-I (Supplementary Figure 1A, 1B, and 1C). The increase of mRNA levels of TLR3, MDA5 and RIG-I after treatment with poly(I:C) was suppressed by specific knockdown of each gene at a level of 60%, 40% and 70%, respectively (Supplementary Figure 1A, 1B and 1C). There was no further suppression of each gene expression by double knockdown of either two genes, indicating that there was no mutual interference of each gene expression.

As for protein expression after siRNA targeting TLR3, MDA5 and RIG-I, there were some difference among these three receptors. While TLR3, as a membrane protein on the endosomes, showed little change after siRNA targeting, MDA5 and RIG-I demonstrated a significant decrease of protein expression after siRNA targeting (Supplementary Figure 2A, 2B and 2C). Similar to mRNA expression, double knockdown did not further suppress MDA5 and RIG-I protein expression levels.

siRNA targeting MDA5 revealed down-regulation of p-IRF3, but was not associated with down-regulation of caspase9 and active caspase3. In fact, treatment of NB cells with poly(I:C) was associated with elevated caspase9 and activated caspase3 with subsequent elevated MAVS regardless scramble siRNA or siMDA5 treatment. A similar findings were found in siRIG-I and siTLR3 treatment (Supplementary Figure 3). However, concomitant knockdown of MDA5 and TLR3 were more effective to suppress upregulation of caspase9 and activated caspase 3 triggered by poly(I:C) treatment in NB cells than either single knockdown or other double knockdowns of TLR3, MDA5, and RIG-I. Interestingly, cleaved MAVS and caspase8 were also most significantly decreased in double knockdown of MDA5 and TLR3 (Figure 1B).

Apoptosis of NB cells, measured by PI staining, showed a significant increase after poly(I:C) treatment, particularly in NB cells treated with scramble siRNA and poly(I:C) (Figure 1C). NB cells treated with scramble siRNA alone did not increase NB cell death. Single knockdown of MDA5 and RIG-I showed little effect in rescuing NB cell death activated by scramble RNA+poly(I:C) treatment, while siTLR3 showed a significant though limited effect in rescuing these cells. However, simultaneous targeting TLR3 and MDA5 with siRNA showed most effective to rescue poly(I:C) or scramble RNA+poly(I:C)-induced NB cell death (Figure 1C).

### Over-expression of MDA5 suppressed colony formation and sensitized poly(I:C) induced cell death in Fadu cells, but had little effect on the survival of SK-N-BE cells

**Figure 2:**
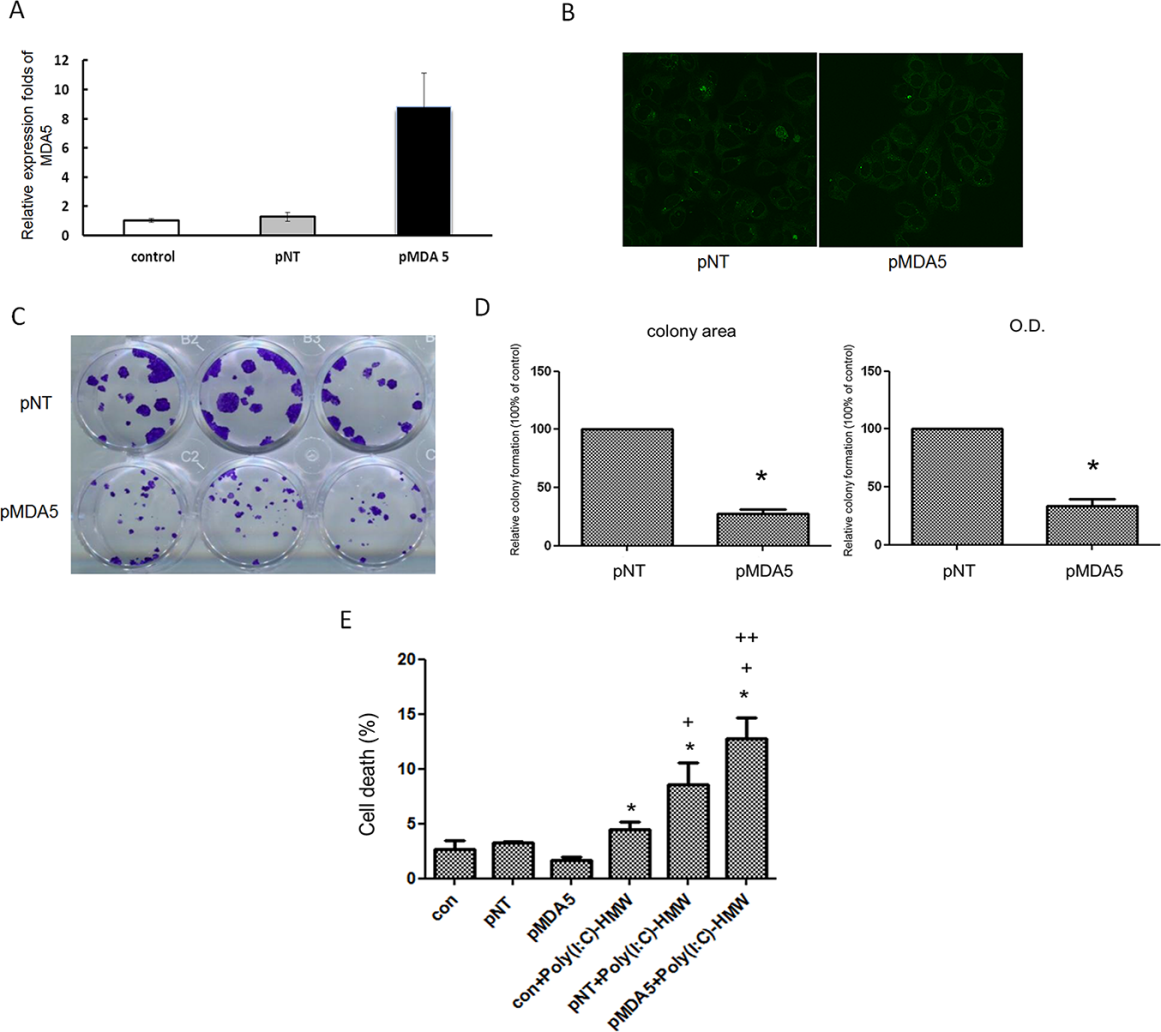
Over-expression of MDA5 in FaDu cells resulted in significantly less colony formation and increased susceptibility to poly(I:C) **A.** FaDu cells was transfected with either GFP plasmid (pNT) or MDA5 expression plasmid (pMDA5) using Lysovec reagent (InvivoGen). Real-time quantitative RT-PCR revealed more than 8-fold increase of MDA5 mRNA levels in pMDA5 transfected cells. **B.** Green fluorescence in pNT and pMDA5 transfected cells confirmed successful transfection. **C.** Significantly smaller colony formation in pMDA5 than pNT transfected cells after 4 weeks of transfection. **D.** The quantitative difference in the colony formation was compared by either colony area or OD value. *p < 0.05 vs. pNT. **E.** Trypan blue exclusion assay showed a significantly higher percentage of cell death in pMDA5 transfected cells after treatment with poly(I:C). *p < 0.05 vs. pNT, +*p* < 0.05 vs. con+poly (I:C), ++*p* < 0.05 vs.pNT+poly(I:C).

Ectopic or over-expression of MDA5 was attempted in NB cells including SK-N-DZ, BE(2)-M17 and SH-SY-5Y, but the cells died immediately after transfection with MDA5 expression plasmid. Several other cell lines were also tried, but only FaDu cells that derived from a squamous cell carcinoma of the hypopharynx survived the transfection, which was confirmed by qRT-PCR and fluorescent microscopy (Figure 2A and 2B). Colony formation of FaDu cells after transfection with MDA5 expression plasmid decreased significantly, as compared to the control cells. (Figure 2C and 2D). Furthermore, treatment of MDA5- overexpression FaDu cells with poly(I:C) resulted in significantly more cell deaths than that of wild type cells (Figure 2E).

Over-expression of MDA5 in NB cells was achievable only in SK-N-BE by Tet-inducible MDA5 expression system (Supplementary Methods and Supplementary Figure 4A). Treatment with poly(I:C) in wild type SK-N-BE cells revealed dose-dependent decrease of cell viability (Supplementary Figure 4B). However, treatment of MDA5-overexpressed SK-N-BE cells with poly(I:C) showed no difference in cell viability comparing to the control wild type cells. (Supplementary Figure 4C).

### Mitochondrial but not intracellular reactive oxygen species was significantly decreased in siTLR3 or siTLR3+siMDA5 treated NB cells

**Figure 3:**
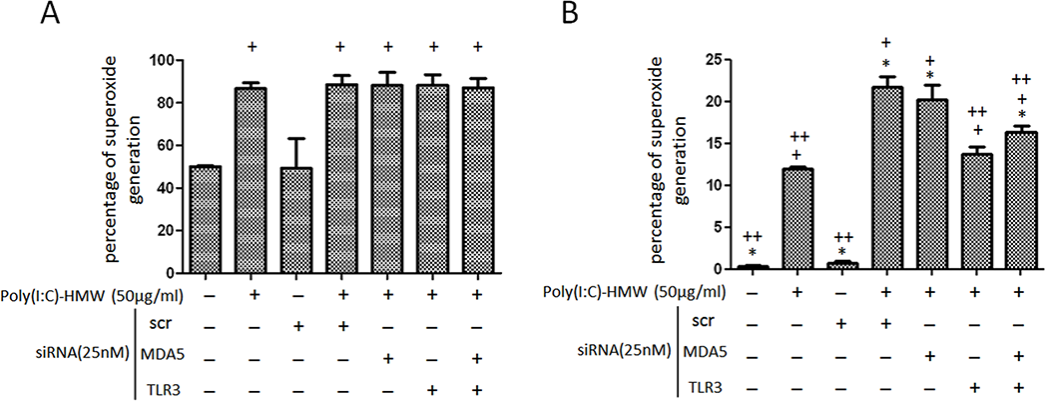
Intracellular and mitochondrial reactive oxygen species changes after single knockdown of TLR3 or concomitant knockdown of TLR3 and MDA5 in NB cells **A.** Intracellular reactive oxygen species (ROS) determined by flow cytometry after cells stained with 6- carboxy-2, 7-dichlorodihydrofluorescein diacetate (DCF-DA) was not affected by single knockdown of TLR3 or concomitant knockdown of TLR3 and MDA5. **B.** Mitochondrial ROS determined by MitoSOX RED™ showed a significant decrease in either siTLR3 or combined siTLR3 and siMDA5 transfected NB cells. scr: scrambled siRNA. *p < 0.05 vs. Poly(I:C), +*p* < 0.05 vs. scr, ++*p* < 0.05 vs. scr+poly(I:C).

Both intracellular and mitochondrial reactive oxygen species (ROS) measured by DCF-DA and MitoSOX RED™, respectively, showed a significant increase after NB cells treated with poly(I:C) (Figure 3A and 3B). The increased intracellular ROS levels were not affected by single knockdown of TLR3 or concomitant knockdown of TLR3 and MDA5 (Figure 3A). However, the increased mitochondrial ROS was significantly suppressed by either single siTLR3 or concomitant siTLR3 and siMDA5 transfection (Figure 3B).

### MDA5 expression in human NB tissues implied a favorable tumor behavior

Since we have shown that double knockdown of TLR3 and MDA5 was most effective to rescue Poly(I:C) induced NB cell death and that high expression of TLR3 in NB tumor tissues carries a favorable patient outcome [4], we would like to further investigate the role of MDA5 expression in human NB tumor behaviors. MDA5 expression in 92 NB tumor samples were evaluate by immunohistochemistry. Positive

**Figure 4:**
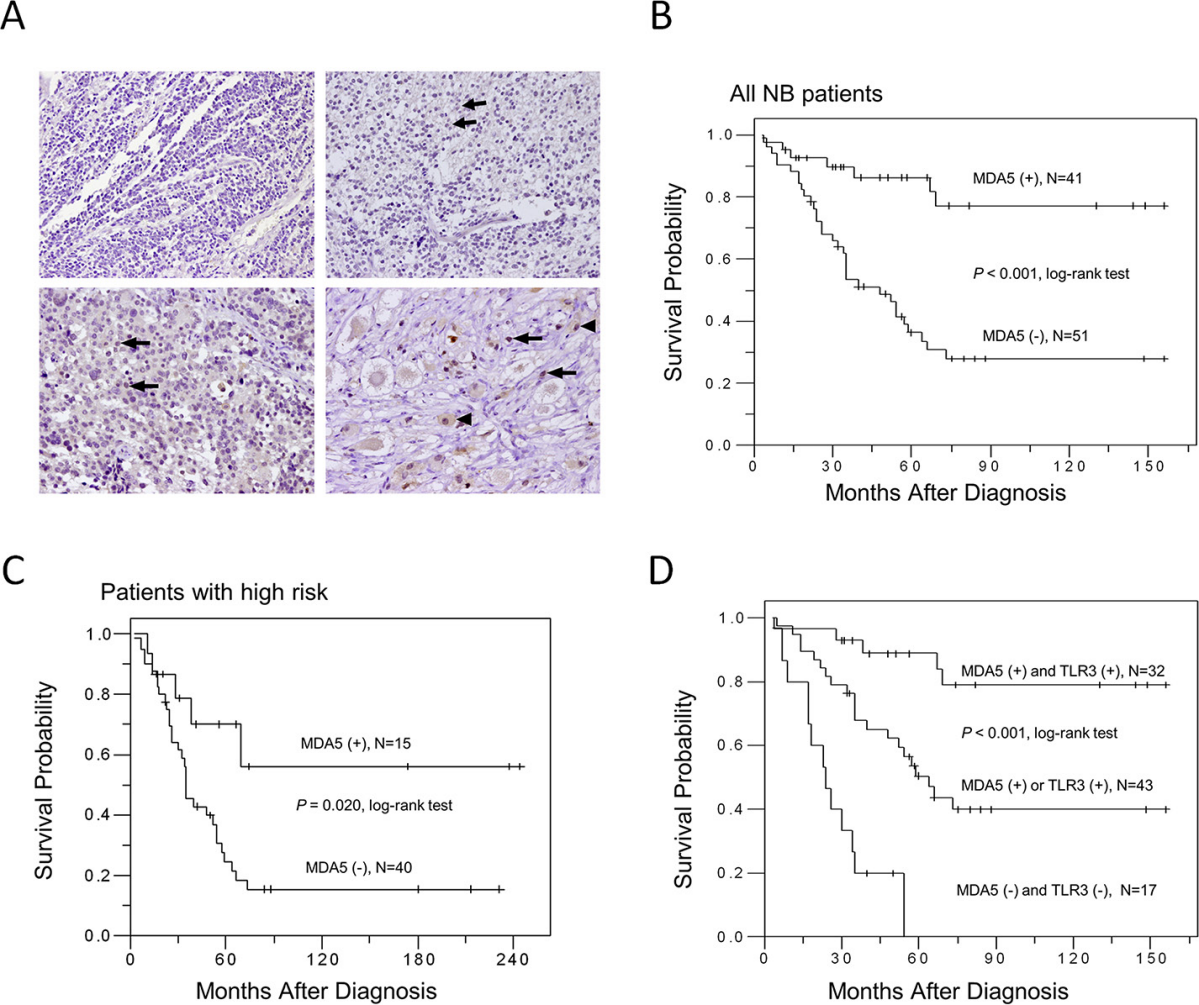
MDA5 expression in human NB tissues and patient survival **A.** Representative pictures of immunohistochemical staining in NB tumors with various degrees of histological differentiation. Arrows and arrow heads indicate positive MDA5 staining. Original magnification 200 ×. **B.** Kaplan-Meier survival analysis in all NB patients according to MDA5 immunostaining. **C.** Kaplan-Meier survival analysis according to the expression of MDA5 in NB patients with high risk. **D.** Kaplan-Meier survival analysis according to the expression of MDA5 and TLR3 in NB patients. *P* value was calculated using log-rank test.

MDA5 immunoreactivity (2+ and 3+) was detected in 41 samples (44.6%). MDA5 immunostaining was negative in neuroblastic cells in undifferentiated NB (Figure 4A, upper left). In differentiating NB, various intensities of MDA5 staining could be found in either the cytoplasm or nucleus (Figure 4A, upper right and lower left, arrows) of the neuroblastic cells. In ganglioneuroblastoma, strong nuclear staining in the neuroblastic cells, as well as nuclear and cytoplasmic staining in ganglion cells was found (Figure 4A, lower right, arrows and arrow heads). No stromal cells demonstrated MDA5 staining. The results suggested that MDA5 expression in NB tissues may indicate a sign of histological differentiation.

Further analysis of the relationship between MDA5 immunostaining and other clinicopathological characteristics of NB, we found that in addition to histological grade of differentiation, positive MDA5 staining also strongly correlated with younger age at diagnosis (*P* = 0.044, χ^2^ test), early clinical stages (*P* = 0.001, χ^2^ test), favorable histology (*P* = 0.007, χ^2^ test), and MYCN non-amplified (*P* = 0.001, χ^2^ test) (Table 1).

**Table 1:** MDA5 expression and clinicopathologic and biologic characteristics of neuroblastomas

	Cases	Positive MDA5 expression (%)	*P* value
Sex			
Male	60	26 (43.3)	0.827
Female	32	15 (46.9)	
Primary tumor site			
Adrenal	61	26 (42.6)	0.660
Extra-adrenal	31	15 (48.4)	
Age at diagnosis			
≤ 1.5 year	28	17 (60.7)	0.044
> 1.5 year	64	24 (37.5)	
Clinical stage			
1, 2, 4S	28	20 (71.4)	0.001
3, 4	64	21 (32.8)	
Tumor histology			
Undifferentiated NB	31	6 (19.4)	< 0.001
Poorly differentiated NB	33	17 (51.5)	
Differentiating NB	12	4 (33.3)	
GNB	16	14 (87.5)	
Shimada histology			
Favorable	48	28 (58.3)	0.007
Unfavorable	44	13 (29.5)	
MYCN			
Amplified	22	3 (13.6)	0.001
Non-amplified	70	38 (54.3)	

To explore the significance of MDA5 expression on patient survival, Kaplan-Meier analysis was performed and showed that patients with MDA5-positive tumors had a better predictive 5-year survival rate than those with MDA5-negative tumors (*P* < 0.001, log-rank test; Figure 4B). Univariate analysis showed that in addition to MDA5 expression, age at diagnosis, clinical stage, *MYCN* status, and Shimada classification could also serve as a prognostic factor for NB (Table 2). Multivariate analysis further revealed that *MYCN* status and negative MDA5 expression remained independent prognostic factors for poor survival (Table 2). To further evaluate the significance of MDA5 expression in prognostic discrimination, the impact of MDA5 expression on survival rate was analyzed according to patients' risk grouping. Both patients with low and intermediate risk had excellent prognosis regardless the MDA5 status. Nevertheless, positive MDA5 expression predicted a better survival probability for NB patients with high risk (*P* = 0.020, log-rank test; Figure 4C). To explore the possible synergistic effect of MDA5 and TLR3 expression on patient survival, Kaplan-Meier analysis according to the expression of MDA5 and TLR3 was performed. The results revealed that patients with double positive staining of MDA5 and TLR3 had the most favorable clinical outcome, while patients with single positive staining of MDA5 or TLR3 had intermediate survival probability, and patients with double negative staining of MDA5 and TLR3 had the poorest survival probability (*P* < 0.001, log-rank test; Figure 4D).

**Table 2:** Clinicopathologic and biologic factors affecting survival rate

Variable	Univariate analysis			Multivariate analysis		
	RR	95% CI	*P* value	RR	95% CI	*P* value
Age at diagnosis: > 1.5 year versus ≤ 1.5 year	4.012	1.895 – 8.493	< 0.001	2.175	0.607 – 7.792	0.233
Clinical stage: advanced (3, 4) versus early (1, 2, 4S)	10.686	3.842 – 29.719	< 0.001	2.376	0.527 – 10.716	0.260
MYCN: amplified versus non-amplified	3.723	2.195 – 6.315	< 0.001	2.191	1.120 – 4.286	0.022
MDA5 expression: negative versus positive	4.718	2.080 – 10.705	< 0.001	2.811	1.127 – 7.013	0.027
Histology: unfavorable versus favorable	3.604	2.037 – 6.376	< 0.001	1.353	0.680 – 2.695	0.389

The results of the above histological studies were further supported by quantitative measurement of MDA5 mRNA expression in the tissue, which demonstrated that NB tumor tissues with MDA5 mRNA expression levels higher than median value showed a higher percentage of positive MDA5 immunostaining (Supplementary Table S1). Furthermore, higher MDA5 mRNA expression in tumors also correlated with younger age at diagnosis, early clinical stages, and favorable Shimada histology (Supplementary Table 1). Survival analysis also confirmed that higher MDA5 mRNA expression predicted a better 5-year survival (Supplementary Figures 5 and 6).

## DISCUSSION

MDA5 and RIG-I are cytosolic receptors responsible for the detection of viral RNA, which have been expected to respond to synthetic dsRNA— poly(I:C) similar to TLR3 in NB [3]. Yet, it has been shown that MDA5 and RIG-I may recognize different types of dsRNAs: MDA5 recognizes poly(I:C), and RIG-I detects *in vitro* transcribed dsRNAs [14, 15]. It is plausible that cytosolic MDA5 may possibly work together with endosome TLR3 to response to poly(I:C) treatment in NB cells. Indeed, in this study we found that while single knockdown of either MDA5 or RIG-I alone fails to suppress poly(I:C)-induced NB cell death, concomitant knockdown of MDA5 and TLR3 was effective to rescue poly(I:C)-induced NB cell death. Importantly, in consistence with the *in vitro* studies, MDA5 and TLR3 expression in tumor tissues also showed a synergistic effect on NB patient survival. The complementary roles of TLR3 and MDA5 have also been demonstrated in other studies with melanoma and NK cells [16, 17]. These lines of evidence strongly support the potential role of targeting innate immune system in the treatment of NB.

In the 6 NB cell lines tested, although all of them showed a variable level of TLR3 expression, none of them showed expression of MDA5 and RIG-I. After treatment with high or low molecular weight poly(I:C), two cell lines (SK-N-AS and SK-N-FI, both are *MYCN* non-amplified) showed increased expression of MDA5 and RIG-I as well as cell apoptosis. Forced expression of MDA5 by plasmid transfection in NB cells induced a rapid cell death even without poly(I:C) treatment. Similar results were also observed in RIG-I transfection experiments (data not shown). It has been shown that RIG-I and MDA5, containing two N-terminal CARD domains and a C-terminal helicase domain, which may trigger DNA fragmentation in response to a small amount of apoptotic cell substrates or active caspase-3 [8]. This finding may explain the instability of the cells transfected with MDA5 or RIG-I, which ultimately leaded to cell death. Over-expression of MDA5 was achievable only in SK-N-BE by Tet-inducible MDA5 expression system. However, this system was hampered by inhibition of cleavage of caspase-3 when exposed to inducing ligand doxycycline and thus interfered the interpretation of the results of cell death [18]. In this study, we have also observed no significant expression of cleavage of caspase-3 (data not shown), which can explain no significant decrease of cell viability when treating MDA5-overexpressed SK-N-BE cells with poly(I:C).

FaDu cells, derived from a squamous cell carcinoma of the hypopharynx, however, could survive from the MDA5 transfection without using Tet-inducible gene expression system. MDA5 over-expressed FaDu cells become sensitive to poly(I:C) induced cell death. Obviously there exists a differential tolerance to MDA5 expression between different cell types. However, more studies are required to verify the surmise.

We found that siTLR3 could rescue NB cells from poly(I:C) induced cell death and that this rescue effect of siTLR3 could be improved by additional suppression of MDA5. In addition, there was no any cell rescue effect by concomitant suppression of MDA5 and RIG-I. These lines of evidence suggest that TLR3 may play a key role in the innate immune mediated NB cell death, while MDA5 may serve a major complementary role to TLR3 in this pathway. Given the finding that MDA5 and RIG-I are absent in all NB cells, a defective innate immune system could be critical for the tumorigenesis of NB.

MDA5 has been a well-known tumor suppressor since it was first identified [6, 8, 19]. Here we showed that high MDA5 expression in NB tumor tissues did correlate well with differentiated tumor histology as well as other favorable clinical and biological factors. Survival analysis revealed that high MDA5 expression in NB tissues predicted a favorable patient prognosis. These findings supported that MDA5 could play as a tumor suppressor in NB. On the other hand, although RIG-I has been recognized as a tumor suppressor in several human cancers including ovarian cancer, leukemia and hepatocellular carcinoma [7, 20, 21], a dual role of RIG-I in human head and neck squamous cell carcinoma has been observed [22]. Upon treatment of cells with viral dsRNA, a low level of RIG-I activation increases host cell survival, whereas a higher level of RIG-I activation leads to cell apoptosis [22]. Furthermore, opposing roles of RNA receptors TLR3 and RIG-I in the inflammatory response to dsRNA has also been shown in a Kaposi's sarcoma cell line SLK [23]. While TLR3 mediated inflammatory response to poly(I:C), siRIG-I resulted in enhanced chemokine release, in a TLR3 pathway-dependent manner [23]. The role of RIG-I in our NB model may be similar to that in SLK. Our results showed that while siRIG-I enhanced poly(I:C) induced apoptosis, further knockdown of TLR3 could reduce the apoptosis rate.

In addition to anti-viral signaling through induction of type I IFN, MDA5 and RIG-I also induced proapoptotic BH3-only protein expression and human melanoma cell death [6]. Here we further showed that upon treatment with high or low molecular weight poly(I:C), there was upregulation of MDA5 and RIG-I with increased expression of caspase9 and cspase3 in NB cells, which was similar to the activation of TLR3 [3]. Yet, knockdown of MDA5 and RIG-I was accompanied by a decrease of p-PKR and p-IRF3 but not a decrease of MAVS, caspase 3, caspase 9, and apoptosis. The above findings support the notion that multiple signaling pathways may be involved in the innate immune mediated apoptosis process [24]. On the other hand, MAVS is known to connect mitochondria with innate immunity. RNA binding induces MDA5 to activate MAVS through interactions between the caspase recruitment domains (CARDs) on these two proteins [25, 26]. It has been shown that MAVS is cleaved during apoptosis after caspase activation [27]. The results of our study echoed these observations by showing that poly(I:C) treatment induced cleavage of MAVS, which was attenuated when either TLR3 or both TLR3 and MDA5 were suppressed, leading subsequently to decreased caspase9 and capase3 expression, mitochondrial ROS production, and NB cells apoptosis.

In this study, we found significant increase in both intracellular (or general) and mitochondrial ROS in poly(I:C)-treated NB cells. However, only increased mitochondrial ROS, but not intracellular ROS, was significantly suppressed by either single siTLR3 or concomitant siTLR3 and siMDA5 transfection. It was consistent with the report that docosahexaenoic acid (DHA)-induced MAPK activation and apoptosis was associated with mitochondrial ROS overproduction and malfunction, and that ROS inhibition remarkably reversed these effects of DHA [28]. It also echoed the findings that ROS induction by a quinone-containing natural product shikonin was of mitochondrial origin, which then induced apoptosis in glioma cells [29].

Despite there was significant increase of TLR3 mRNA expression after poly(I:C) treatment of SK-N-AS cells, the protein levels showed no significant change, which was contrary to the expression of MDA5 and RIG-I. Similar findings were shown in 9-cis-retinoic-acid (9cRA) treatment of SK-BR-3 breast cancer cells, which revealed no significant change of TLR3, MDA5 and PKR, but significant up-regulation of RIG-I. However, poly(I:C) could synergize with 9cRA to up-regulate TLR3, RIG-I, MDA5 and PKR, leading to apoptosis of SK-BR-3 cells [30]. Similar findings were shown in Me 260 cells, which failed to respond to poly(I:C) alone, but IFN-α+ poly(I:C) could induce up-regulation of TLR3 and induced melanoma cell death [31].

In summary, we identify a particular tumor suppressor role for MDA5 in NB. MDA5 may complement TLR3 dependent innate immune mediated NB cell death. In addition, MDA5 expression may predict a favorable outcome in NB patients with a synergistic effect with TLR3. Further elucidating the role of TLR3 and MDA5 expression in NB may not only shed light to the pathogenesis of NB but also potential therapy of NB by targeting innate immune system.

## MATERIALS AND METHODS

### Cell lines and culture

Six human NB cell lines (SK-N-AS, SK-N-FI, SK-N-DZ, IMR-32, BE(2)-M17 and SH-SY5Y) were purchased from the American Type Culture Collection (Manassas, VA, USA). Cell culture reagents were purchased from Invitrogen (Carlsbad, CA, USA). SK-N-AS, SK-N-FI and SK-N-DZ were cultured with Dulbecco's modified Eagle's medium (DMEM) containing L-glutamine; IMR-32 was cultured with Eagle's Minimum Essential Medium (MEM) containing sodium pyruvate; BE(2)-M17 was cultured with a 1:1 mixture of MEM and F-12 medium containing sodium pyruvate; SH-SY-5Y was cultured with a 1:1 mixture of DMEM and F-12 medium. All of the above culture mediums contain 10% (v/v) heat-inactivated fetal bovine serum (FBS), 10 mM nonessential amino acids and antibiotic-antimycotic. Cells were cultured in 5% CO_2_ humidified incubator at 37°C.

### Treatment of human NB cells with poly(I:C)-HMW or poly(I:C)-LMW

1 × 10^6^ cells were seeded in 100-mm culture dishes and treated with 50 μg/ml of polyinosinic:polycytidylic acid high molecular weight [poly(I:C)HMW; Invitrogen] or polyinosinic:polycytidylic acid low molecular weight [poly(I:C)LMW; Invitrogen] in culture medium plus 10% FBS for 24 h. Poly(I:C)HMW has an average size of 1.5–8 kb, while poly(I:C)LMW has an average of 0.2–1 kb.

### Small-interfering RNA targeting MDA5, RIG-I and TLR3

Small-interfering RNA targeting human MDA5 and TLR3 gene (siMDA5 and siTLR3, respectively), and control (scramble) siRNA were purchased from GeneDireX (Las Vegas, NV, USA). The sequences for siMDA5 was 5′-GUAUCGUGUUAUUGGAUUAdTdT-3′; for siRIG-I 5′-AUCACGGAUUAGCGACAAAdTdT-3; for siTLR3, 5′-AAAGGUAGUGGCUUGACAGCUCAGG-3′ and for control siRNA, 5′-UUCUCCGAACGUGUCACGUTT-3′, respectively. MDA5 siRNA was designed according to published guidelines. Gene delivery of siRNA into SK-N-AS was performed using Lipofectamine RNAiMAX (Invitrogen, Carlsbad, CA) following the manufacturer's protocol. 5 × 10^6^ cells were seeded in 100-mm culture dishes 24 h before transfection. For single knockdown, 25 nM siRNA in 7.5 μl DEPC water (tube 1) and 20 μl of Lipofectamine RNAiMAX reagent (tube 2) were separately diluted in 500 μl Opti-MEM (Invitrogen, Carlsbad, CA) without serum, and then mixed and incubated for 15–20 min at room temperature. For double knockdown, 25 nM of two different siRNAs in 7.5 μl (tube 1) and 20 μl of Lipofectamine RNAiMAX reagent (tube 2) were separately diluted in 500 μl Opti-MEM without serum, and then mixed and incubated for 15–20 min at room temperature. The mixture was put into culture dishes containing NB cells for 6 h and the culture medium was replaced and transfection continued for another 18 h (for a total of 24 h). The cells were then incubated with 50 μg/ml poly(I:C)HMW for 24 h before analysis.

### Over-expression of MDA5 in NB and FaDu cells

The MDA5 expression plasmid [IFIH1 human cDNA ORF Clone] and control plasmid (pMV6-AC-GFP) were obtained from OriGene Technologies (Rockville, MD). Stable transfection of cells with MDA5 expression plasmid was performed by culturing BE(2)-M17 cells and SH-SY-5Y cells. For transfection, 2 × 10^6^ cells were seeded in 60-mm culture dishes one day before transfection. On the second day, 8 μg plasmid DNA (tube 1) and 20 μl of Lipofectamine 2000 reagent (tube 2) were diluted in 500 μl Opti-MEM (Invitrogen, Carlsbad, CA) without serum in separate tubes. Combine and mix the diluted plasmid DNA and Lipofectamine 2000 reagent gently, then incubate for 15–20 minutes at room temperature. After incubate, add the mixture into culture dishes containing cells. Mix gently by rocking the plate back and forth. After 6 h, the medium was replaced with culture medium containing 10% FBS. Approximately 48 h after transfection, the transfected cells were selected with G418 (800 μg/ml).

After failed NB cell transfection with MDA5 expression plasmid, we shifted to use FaDu cells. FaDu cell line was derived from a squamous cell carcinoma of the hypopharynx and was obtained from the American Type Culture Collection (Manassas, VA, USA). The cells were incubated in Minimum Essential Media (MEM) medium containing 4.5 g/ml glucose (Invitrogen Life Technologies, Carlsbad, CA, USA) supplemented with 1 mM sodium pyruvate (Invitrogen Life Technologies), 10% fetal bovine serum (FBS; Invitrogen Life Technologies). FaDu (3 × 10^5^ cells in six-well plates) was transfected with either GFP (green fluorescent protein) plasmid (pMV6-AC-GFP) as a non-targeting control or MDA5 expression plasmid (IFIH1 human cDNA ORF Clone) using Lysovec reagent (InvivoGen). At 48 h after transfection, we aspirated the medium and replaced it with fresh medium containing 500 *μ*g/ml selective antibiotics G418. This selection medium was refreshed every 2 days. We monitored the cells daily and observed the percentage of surviving cells stably expressing MDA5 or control plasmids. At approximately 4 weeks following transfection, stable cells were visualized by fluorescent microscopy and harvested for qRT-PCR (quantitative real-time PCR) analysis.

### Colony formation assay

Fadu cells transfected with MDA5 expression plasmid or control plasmid were trypsinized and replated at 100 cells per 12 well plate with complete medium containing G418 (500 μg/ml). The medium containing G418 was replaced every two days. After 20 days, cells were fixed with cold methanol and stained with 0.5% crystal violet solution. The area of colonies was calculated using Image J software.

### Trypan blue exclusion assay

Cell death was analyzed by staining cells with Trypan blue (BioWhittaker, MD, USA). Cells were harvested and suspended in 2 ml complete medium, then 10 μl of cell suspension was mixed with 10 μl of 0.4% Trypan blue. Stained cells and unstained cells were counted with cell counter under microscope, which was further confirmed by FACScalibur flow cytometer using CellQuest software.

### Western blot analysis

After treatment, cells were lysed with PRO-PREP™ Protein Extraction Solution (iNtRON Biotechnology; Seoul, Korea) and protein concentrations were measured with the bicinchoninic acid assay (Bio-Rad; Richmond, CA) using bovine serum albumin as the standard. Crude proteins (30 μg) were separated in 10–15% sodium dodecyl sulfate polyacrylamide electrophoresis gels and transferred to nitrocellulose membranes. The membranes were immunoblotted overnight at 4°C with each primary antibody at the indicated dilution. The primary antibodies included MDA5 (D74E4; Cell Signaling Technology, Danvers, MA, USA), RIG-I (D14G6 ; Cell Signaling Technology, Danvers, MA, USA), TLR3 (Abcam Inc., Cambridge, MA, USA), MAVS (Santa Cruz Biotechnology), cleaved caspase 8 (D391, Cell Signaling Technology, Danvers, MA, USA), cleaved caspase 9 (Asp330, Cell Signaling Technology, Danvers, MA, USA), caspase3 (Cell Signaling Technology, Danvers, MA, USA), cleaved caspase-3 (Asp175; Cell Signaling Technology, Danvers, MA, USA), p-PKR (pT451; EPITOMICS), PKR (N-term; EPITOMICS), p-IRF3 (pS386; EPITOMICS), IRF3 (N-term; EPITOMICS), and β-actin (Millipole, Billerica, MA, USA). Membranes were washed three times and incubated with a horseradish peroxidase-conjugated secondary antibody for 60 min at room temperature. Protein-antibody complexes were visualized with an electrochemiluminescence western blotting detection and analysis system (Amersham Pharmacia Biotech, Uppsala, Sweden), and blots were exposed to film. Signals were quantified by densitometric analysis.

### Flow cytometry analysis of apoptosis and reactive oxygen species

The percentage of apoptotic cells was determined by flow cytometry analysis following propidium iodide staining (PI; BD Biosciences, San Jose, CA). For PI staining, 1 × 10^6^ cells were seeded in 60-mm culture dishes. After siRNA transfection for 24 hr, the cells were then cultured in medium with or without 50 μg/ml poly(I:C)HMW for 24 h. Cells were harvested and incubated in 100 μl PBS containing 5 μl PI (50 μg/ml) for 15 min at room temperature. Samples were analyzed using a FACS (BD Biosciences, San Jose, CA).

Intracellular and mitochondrial reactive oxygen species were determined by flow cytometry, after cells were stained with 6- carboxy-2, 7-dichlorodihydrofluorescein diacetate (DCF-DA) (Sigma) fluorescent probe and MitoSOX RED™ kit (Molecular probe, Invitrogen), respectively. The procedures for each measurement followed the protocol provided by the manufacturers. The fluorescence was analyzed on FACScalibur flow cytometer (BD Biosciences) using CellQuest software.

### Patients and tumor samples

From January 2000 to December 2012, 92 patients with NB who were treated and had complete follow-up were enrolled in the study. The study was approved by Institutional Ethics Committee (100–3966C issued by IRB of Chang Gung Medical Foundation on February 1, 2013). The median age at diagnosis was 2.2 years (range 0–11.5 years). The male to female ratio was 60:32. Adrenal gland was the most common primary tumor site (61/92). Tumor histology was categorized into four types according to the criteria of the International Neuroblastoma Pathology Classification (INPC) [32, 33] including: undifferentiated NB (UNB), poorly differentiated NB (PDNB), differentiating NB (DNB), and ganglioneuroblastoma (GNB), intermixed. The GNB, nodular subtype, was classified into UNB, PDNB, or DNB according to the morphologic features of the NB nodules since these nodules determine the clinical outcome. For prognostic analysis, tumors were further classified as either favorable or unfavorable according to the mitosis-karyorrhexis index and the patient age at diagnosis based on the criteria of the INPC [32, 33]. Tumor staging was classified according to the International NB Staging System [34]. *MYCN* status was evaluated using chromogenic *in situ* hybridization analysis of formalin-fixed paraffin-embedded tissues [35]. Based on risk classifications of the Children's Cancer Group (CCG), patients were classified into low-, intermediate-, and high-risk groups and risk-directed therapies were applied accordingly [36]. The median follow-up after diagnosis was 38 months (range of 1–156 months), and the overall predictive 5-year survival rate for this cohort was 55.6%.

### Immunohistochemical staining

The tissue sections were sliced from the paraffin blocks and placed onto the silane-coated slides. After deparaffinization, the sections were treated with 3% H_2_O_2_ for 10 minutes, followed by antigen retrieval with microwave in 10 mM citrate buffer pH6.0 for 15 min to unmask the epitopes. Then the sections were incubated with diluted primary antibodies directed against melanoma differentiation-associated protein 5 (MDA-5, 1:50 dilution; ProSci Inc., Poway, CA, USA) at room temperature for 1 hour and rinsed in PBS. Thereafter, a polymerized reporter enzyme staining system (ImmPRESS™ universal reagent, Vector Laboratories, Burlingame, CA, USA) was applied for 30 min according to the manufacturer's recommendation. Finally, the signals were visualized by treatment with ImmPACT™ DAB peroxidase substrate (Vector Laboratories) and mounted. The staining method for TLR3 has been described elsewhere [4]. The staining intensity was scored as 0: no staining, 1: faint or weak staining, 2: moderate staining, and 3: strong staining. All the sections were initially scored by two pathologists (Chao-Cheng Huang; Hong-Lin He) who did not know the clinical data, respectively. For the cases with discordant results, the two pathologists read the slides again together to get a concordance. Both scores 0 and 1 were grouped as negative staining, and both scores 2 and 3 as positive staining for the further statistical analysis since there was a significant difference of survival possibility between these two groups of patients (Supplementary Figure 4).

To further confirm the results of clinical significance of MDA5 expression in NB evaluated by immunohistochemistry, 35 NB fresh frozen tumor tissues were subjected to quantitative RT-PCR analysis of mRNA expression.

### Statistical analysis

All the data present in the figures were representatives of at least triplicate experiments. Data were expressed as mean ± SD. Student's *t*-test was used for between-group comparison while analysis of variance for continuous variables among groups and Mann-Whitney *U*-test for nonparametric data. Survival analysis was done by using the Kaplan-Meier method and the Wilcoxon log-rank test to compare difference between groups. Statistical analysis of histological findings between different NB groups was performed using Fisher's exact test. A *P*-value less than 0.05 was considered statistically significant.

## SUPPLEMENTARY MATERIALS AND METHODS FIGURES AND TABLE


