# Supplementary material for: MDA5 complements TLR3 in suppression of neuroblastoma
Source: Oncotarget. 2015 Jul 9;6(28):24935–46. doi: 10.18632/oncotarget.4511 (PMC4694805; doi:10.18632/oncotarget.4511)
Supplement: Supplementary file 1 [file oncotarget-06-24935-s001.pdf]

SUPPLEMENTARY MATERIALS AND METHODS

Generation of MDA5 inducible expression SK-N-BE cell line

To set up a Tet-inducible MDA5 expression system, SK-N-BE cells were first transfected with the pAS2.V1-TR vector, encoding the tetracycline repressor behind a constitutive CMV promoter by lentivirus transduction system. The drug resistant clones of SK-N-BE-TR were obtained by Puromycin selection (2 μg/ml). The MDA5 genes were cloned into the pcDNA5/TO vectors and then transfected into the SK-N-BE-TR cells by Lipofectamine 2000. After around a 2-month Hygomycine B (400 μg/ml) selection, the antibiotic-resistant clones were achieved and amplified for the following experiments.

Cell viability assay

Cell viability was assessed using the CellTiter 96® AQueous One Solution Cell Proliferation Assay. Cells plated in 96-well plates were treated with different concentrations of poly(I:C) in 100 μl DMEM culture medium with 2% FBS for 24 hr. After treatment, 20 μl of CellTiter 96®AQueous One Solution Reagent was added to each well and incubated at 37°C for 1 hr. Absorbency was measured by a spectrophotometer at the wavelength of 490 nm. All viability assays were repeated there times at least. One way ANOVA was used to determine the significance of difference between means, with p values < 0.05 being considered significant.

SUPPLEMENTARY FIGURES AND TABLE

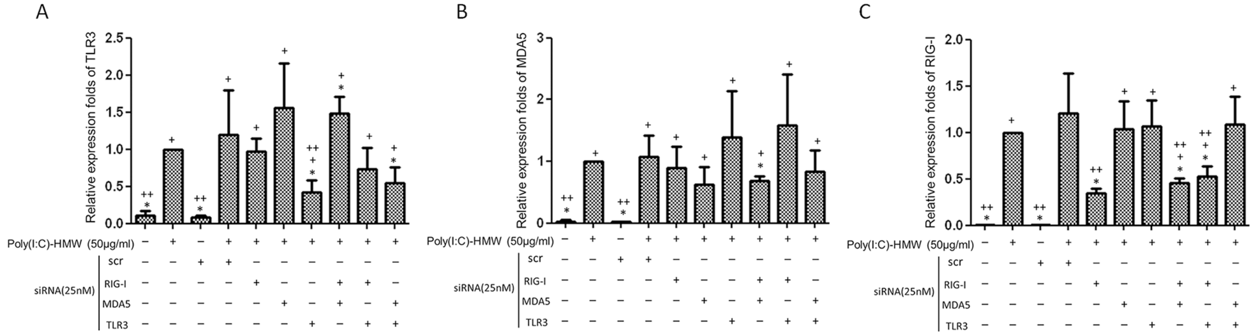

Supplementary Figure 1: mRNA expression levels of TLR3, MDA5, and RIG-I after treatment with poly(I:C) and single or concomitant knockdown of TLR3, MDA5, and RIG-I. A. TLR3, **B.** MDA5, and **C.** RIG-I mRNA expression levels in SK-N-AS cells after treatment with poly(I:C)-HMW and single or concomitant knockdown of TLR3, MDA5, and RIG-I. *p < 0.05 vs. Poly(I:C), +p < 0.05 vs. scr, ++p < 0.05 vs. scr+poly(I:C).

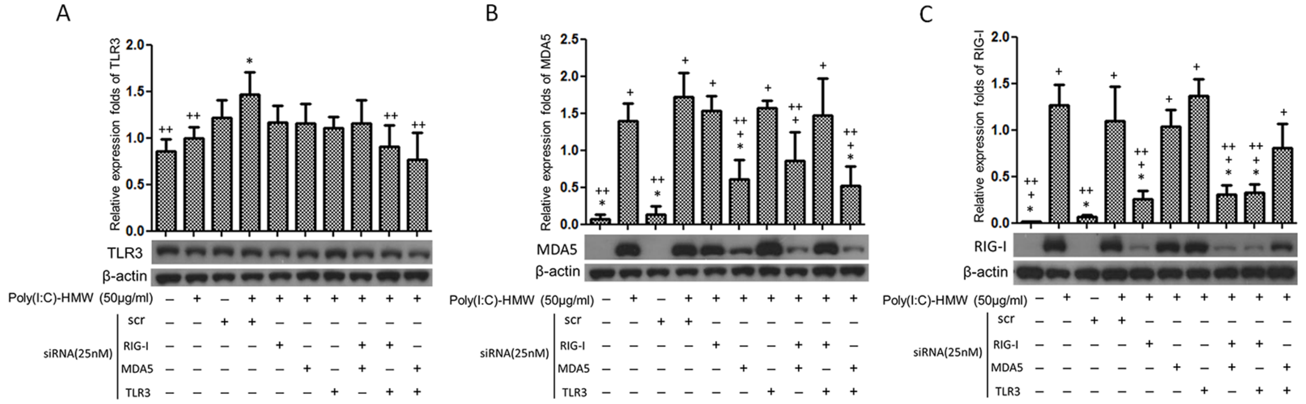

Supplementary Figure 2: Protein expression levels of TLR3, MDA5, and RIG-I after treatment with poly(I:C) and single or concomitant knockdown of TLR3, MDA5, and RIG-I. A. TLR3, **B.** MDA5, and **C.** RIG-I protein expression levels in SK-N-AS cells after treatment with poly(I:C)-HMW and single or concomitant knockdown of TLR3, MDA5, and RIG-I. *p < 0.05 vs. Poly(I:C), +p < 0.05 vs. scr, ++ p < 0.05 vs. scr+poly(I:C).

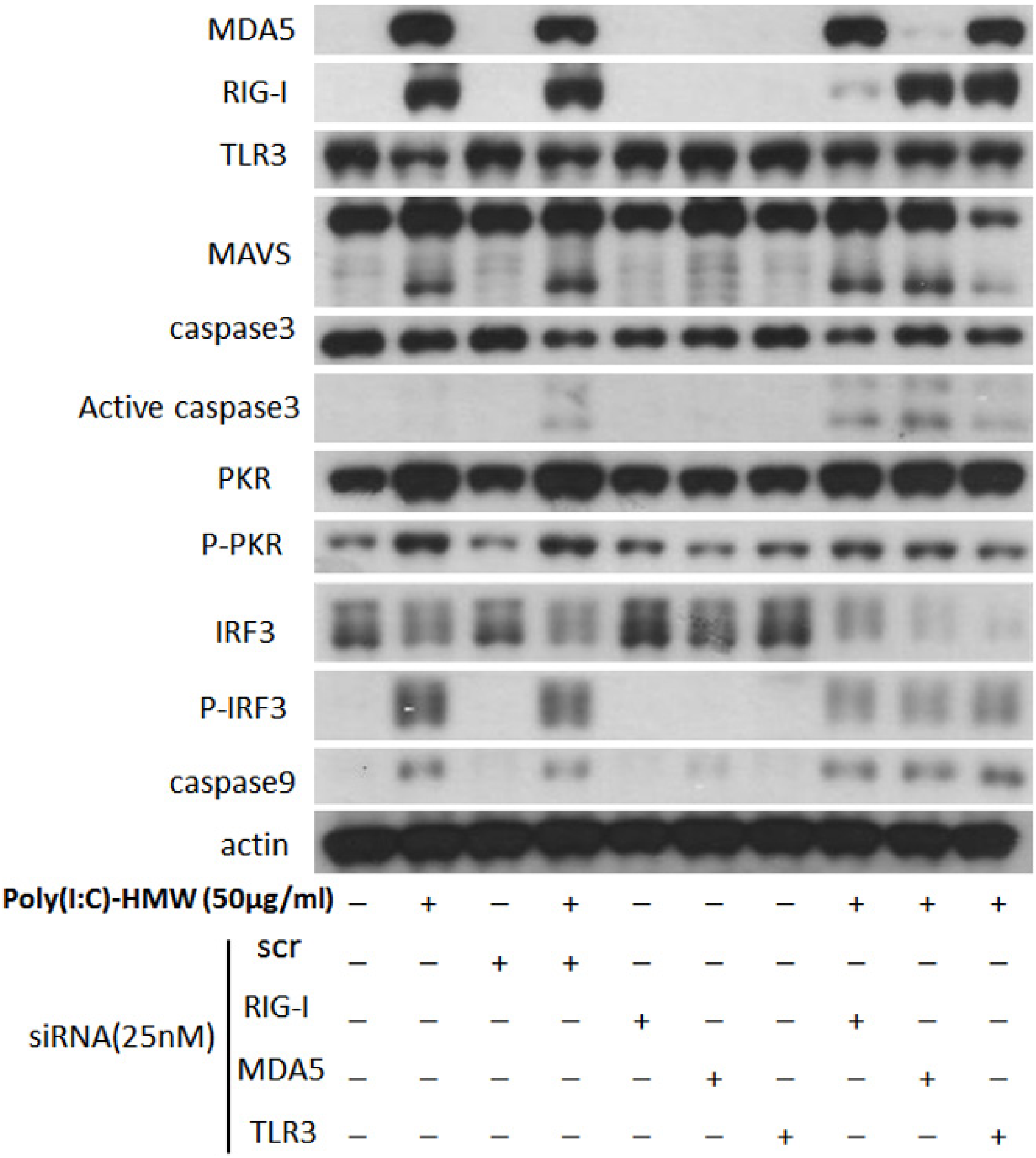

Supplementary Figure 3: Expression of PKR, IRF3, MAVS, caspase 9 and caspase 3 in SK-N-AS after poly(I:C) treatment and siRNA targeting MAD5, RIG-I or TLR3. Treatment of SK-N-AS cells with poly(I:C)-HMW [or simply poly(I:C)] resulted in significant increase of p-PKR, P-IRF3, MAVS, caspase9 and active caspase3. Although siRNA targeting MDA5, RIG-I and TLR3 was effective to reduce p-PKR, IRF3 and P-IRF3, but only TLR3, but not siRIG-I or siMDA5, could reduce poly(I:C)-induced up-regulation of MAVS and active caspase3.

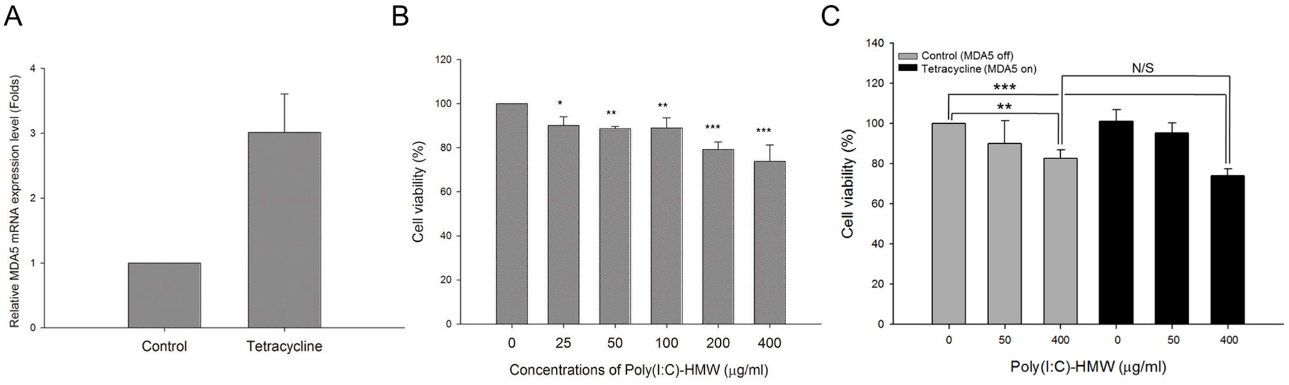

Supplementary Figure 4: A–C. A, MDA5 expression was induced by 1 μg/ml tetracycline for 24 hr. The mRNA expression level of MDA5 was examined by SYBR Green real time PCR with a specific primer; B, SK-N-BE cells were treated with different concentrations of poly(I:C). Cell viability was examined by CellTiter 96® AQueous One Solution Cell Proliferation Assay. Poly(I:C) induced cell death in a dose-dependent manner; C, MDA5 inducible SK-N-BE cells were pretreated with 1 μg/ml tetracycline for 24 hr followed with poly(I:C) treatment for another 24 hr. The effect of MDA5 on poly(I:C)-induced cell death was examined by CellTiter 96® AQueous One Solution Cell Proliferation Assay.

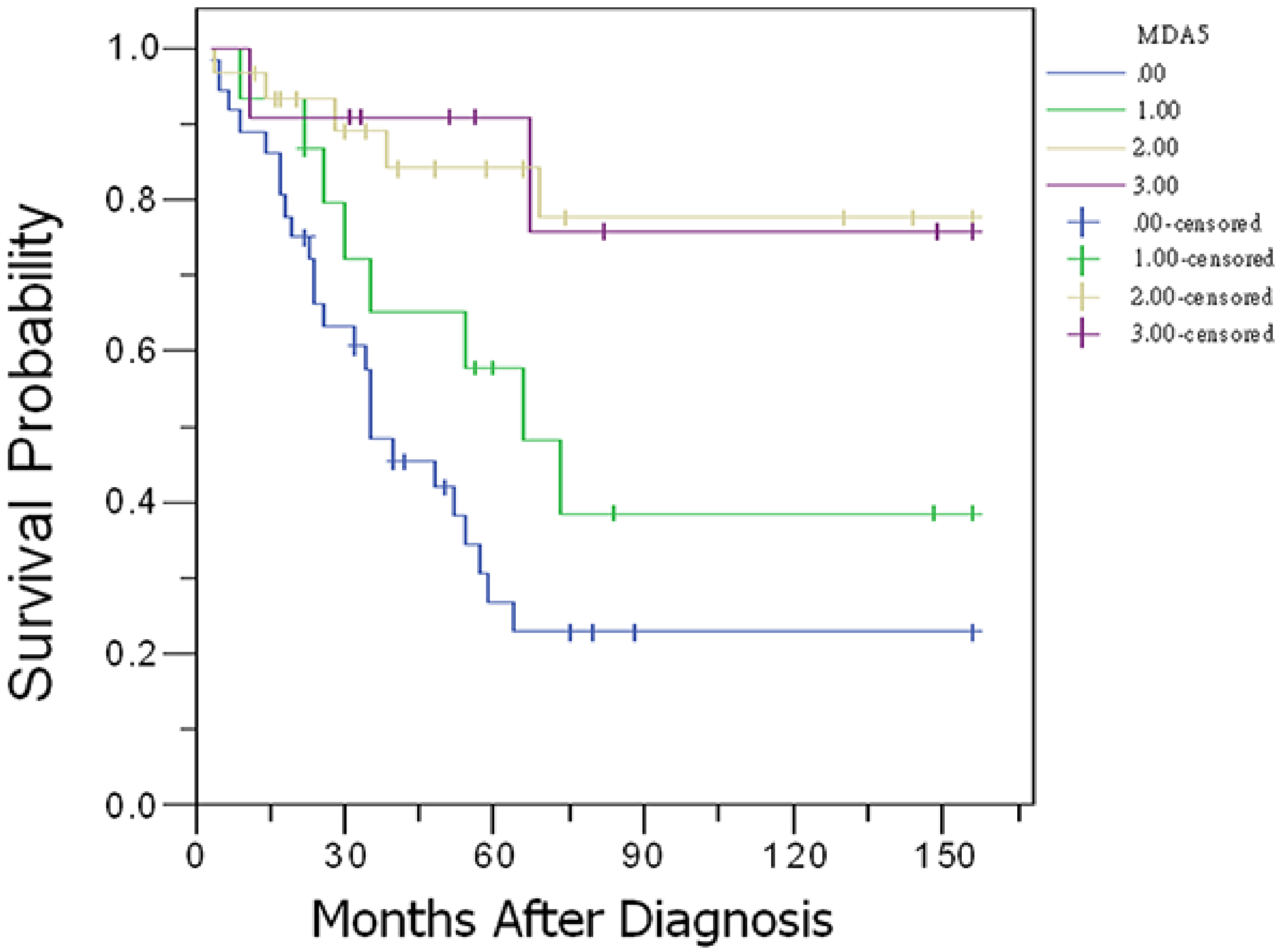

Supplementary Figure 5: Kaplan-Meier survival analysis in all NB patients according to different levels of MDA5 mRNA expression.

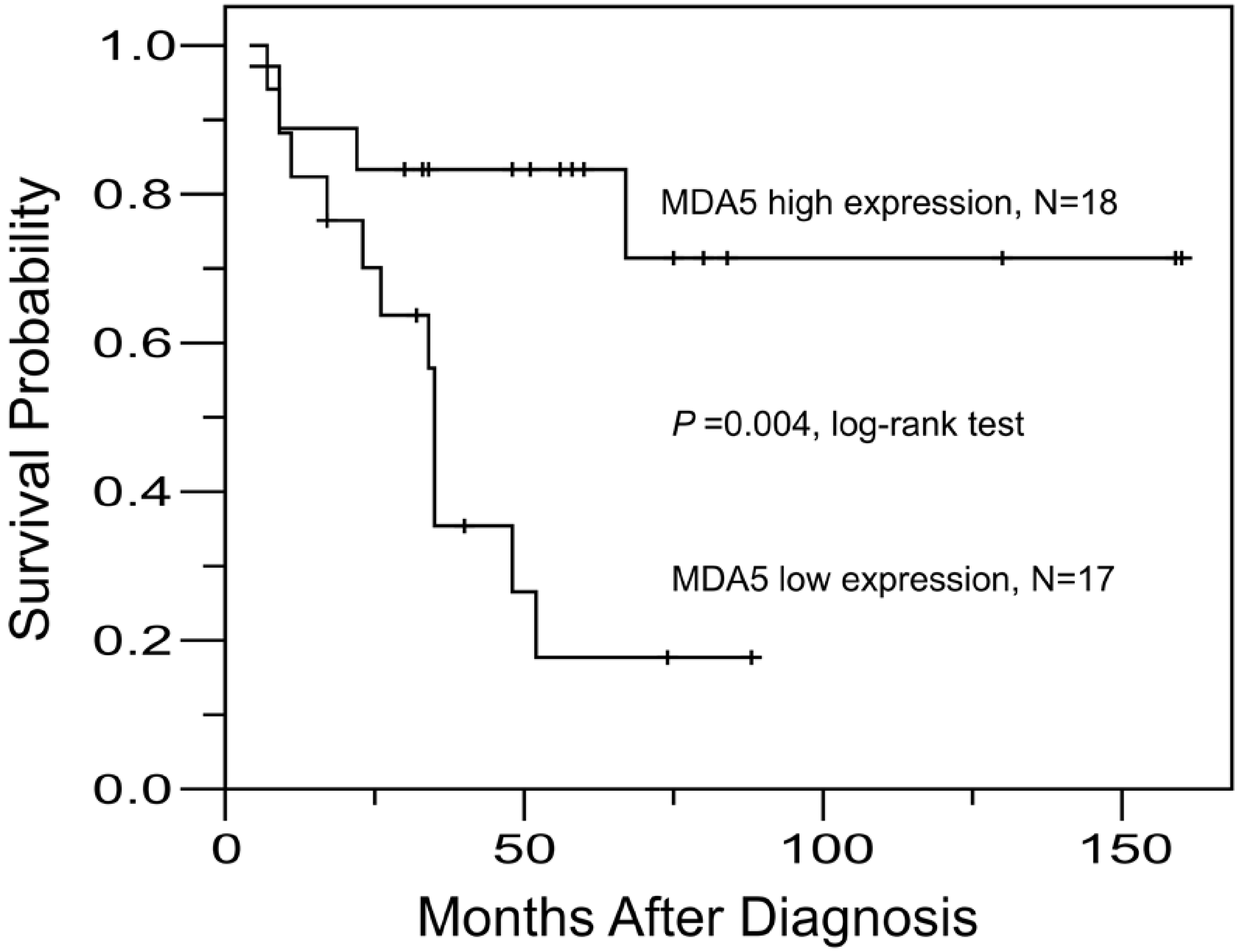

Supplementary Figure 6: Kaplan-Meier survival analysis in all NB patients according to arbitrarily high or low levels of MDA5 mRNA expression.

Supplementary Table 1: MDA5 mRNA expression and clinicobiologic characteristics of neuroblastomas

	Cases	MDA5 high expression (%)	*P* value
MDA5 immunostaining			
Positive	15	12 (80.0)	0.006
Negative	20	6 (30.0)	
Primary tumor site			
Adrenal	24	11 (45.8)	0.471
Extra-adrenal	11	7 (63.6)	
Age at diagnosis			
≤ 1.5 year	12	10 (83.3)	0.012
> 1.5 year	23	8 (34.8)	
Clinical stage			
1, 2, 4S	28	20 (71.4)	0.001
3, 4	64	21 (32.8)	
Tumor histology			
Undifferentiated NB	10	2 20.0)	0.071
Poorly differentiated NB	14	10 (71.4)	
Differentiating NB	5	2(40.0)	
GNB	6	4 (66.7)	
Shimada histology			
Favorable	22	15 (68.2)	0.015
Unfavorable	13	3 (23.1)	
MYCN			
Amplified	9	3 (33.3)	0.264
Non-amplified	26	15 (57.7)	
